# Supplementary material for: Temporal Resistome and Microbial Community Dynamics in an Intensive Aquaculture Facility with Prophylactic Antimicrobial Treatment
Source: Microorganisms. 2020 Dec 13;8(12):1984. doi: 10.3390/microorganisms8121984 (PMC7764744; doi:10.3390/microorganisms8121984)
Supplement: Supplementary file 1 [file microorganisms-08-01984-s001.pdf]

Supporting information:

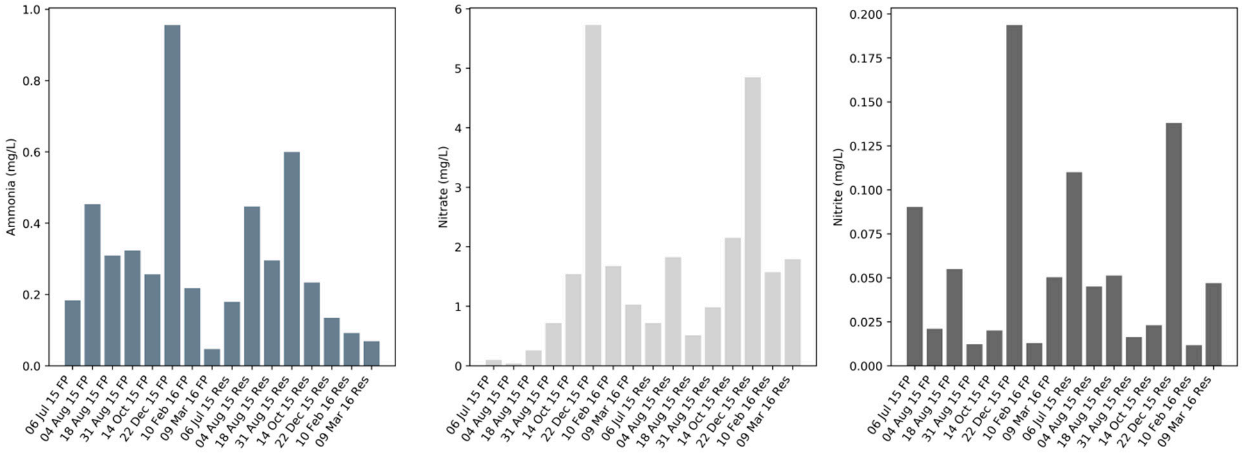

Fig S1 Temporal analysis of ammonia, nitrite and nitrate in the fishpond-3 (FP) and the reservoir (Res) water columns at the Dor aquaculture research station.

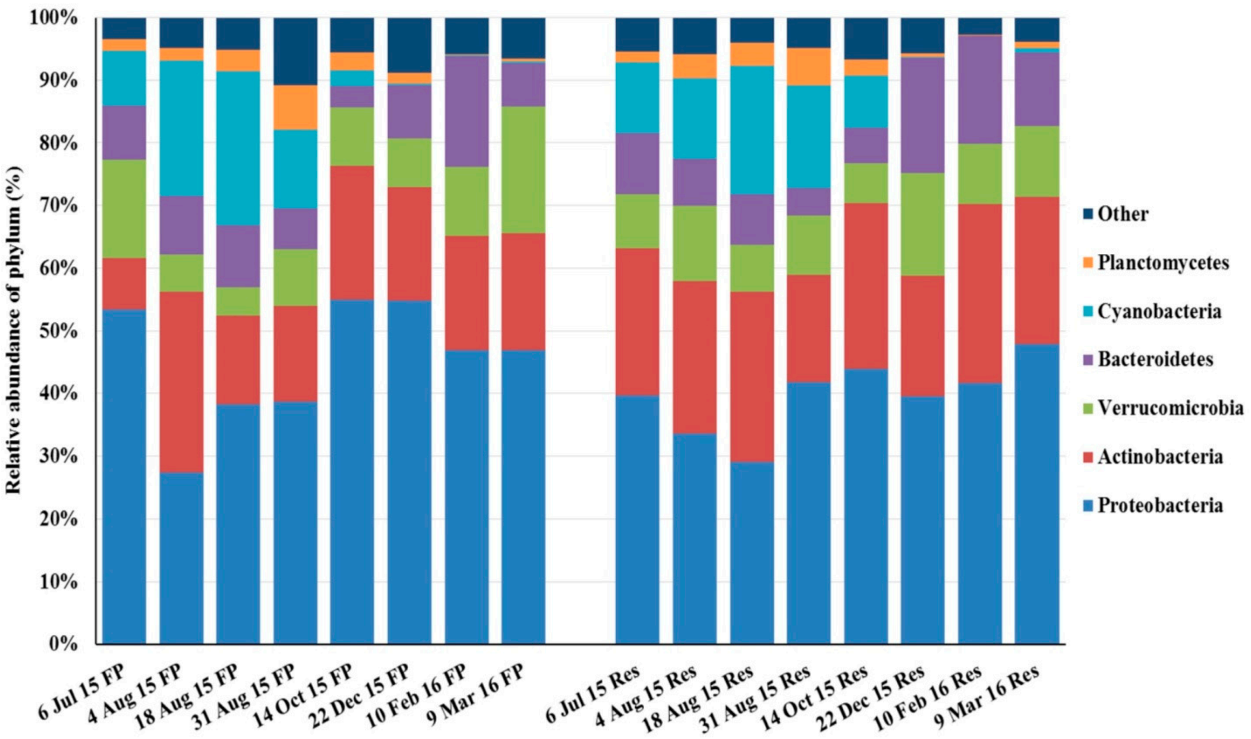

Fig S2 Relative abundance of dominant bacterial phyla in the Dor fishpond-3 (FP) and reservoir (Res) water columns.

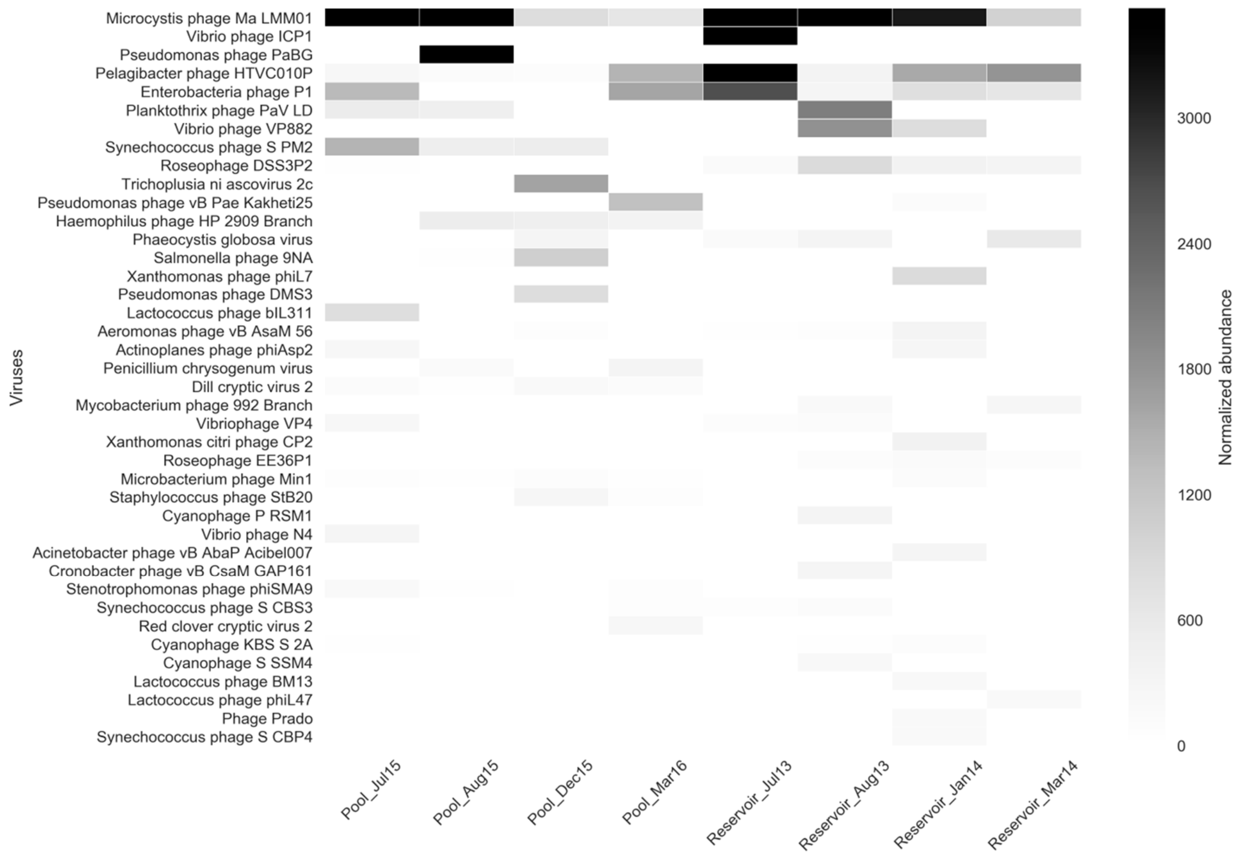

Fig S3 Heat map showing the relative abundance of predominant virusesinfishpond-3 (Pool) and the reservoir, based on shotgun metagenomic data analysed using the highly curated COSMOSID pipeline.

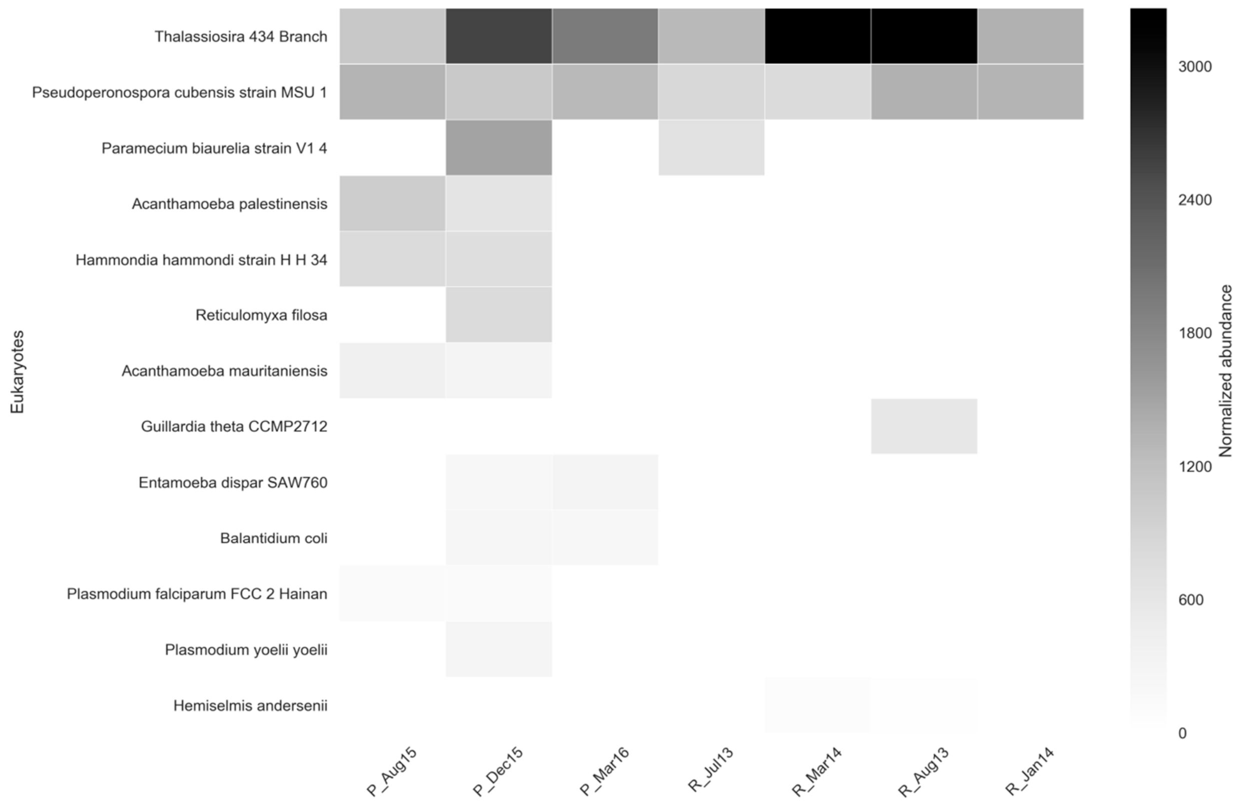

Fig S4 Heat map showing the relative abundance of predominant protistsinfishpond-3 (P) and the reservoir (R), based on shotgun metagenomic data analysed using the highly curated COSMOSID pipeline.

Table S1. Samples and analyses details followed in this study.

**Sampling site**				
Fishpond 3 (FP3) and water storage reservoir (Res), Dor aquaculture research station, Israel				
**Fish breed**				
Silver Carp (*Hypophthalmichthys molitrix*)				
**Total number of samples**				
Fish pond 3: 19 samples and Reservoir: 19 samples				
**Sampling Profile**	**Replicates processed for DNA Extraction**	**Replicates processed for physicochemical analysis**	**Samples processed for Shotgun sequencing**	**Samples processed for qPCR analysis**
July 6th, 2015	2	2	1 (Jul 2015)	1
August 4th, 2015	2	2	0	1
August 18th, 2015	2	2	1 (Aug 2015)	1
August 31st, 2015	3	3	0	1
October 14th, 2015	3	3	0	1
December 22nd, 2015	3	3	1 (Dec 2015)	1
February 10th, 2016	1	1	0	1
March 9th, 2016	3	3	1 (Mar 2016)	1
**Total**	**19**	**19**	**4**	**8**
July 6th, 2015	2	2	1 (Jul 2013)	0
August 4th, 2015	2	2	0	0
August 18th, 2015	2	2	1 (Aug 2013)	0
August 31st, 2015	3	3	0	0
October 14th, 2015	3	3	0	0
December 22nd, 2015	3	3	0	0
February 10th, 2016	1	1	1 (Jan 2014)	0
March 9th, 2016	3	3	1 (Mar 2014)	0
**Total**	**19**	**19**	**4**	**0**

*Water was sampled from the upper 10 cm of the water columnfrom the less disturbed areas of the pool and reservoir

S2.Physicochemical parameters at the study site during sampling

Sampling point	Temp (°C)	pH	Precipitation (mm)	Ammonia (ppm)	Nitrite (ppm)	Nitrate (ppm)	Aquatic sul (ppb)	Aquatic trim (ppb)
6-Jul-15	25.6	8.2	0.0	0.18	0.09	0.10	0.00	9.63
4-Aug-15	27.8	8.0	0.0	0.45	0.02	0.04	4.93	6.97
18-Aug-15	27.8	8.0	0.0	0.31	0.06	0.26	0.13	5.87
31-Aug-15	27.8	7.9	0.0	0.32	0.01	0.72	0.00	6.07
14-Oct-15	23.5	8.1	58.5	0.26	0.02	1.54	0.00	4.53
22-Dec-15	14.7	7.9	52.0	0.96	0.19	5.73	0.00	3.57
10-Feb-16	15.9	7.7	63.7	0.22	0.01	1.68	0.10	1.93
9-Mar-16	17.7	7.7	20.1	0.05	0.05	1.03	0.00	2.83

(Temp: temperature; mm: millimeter; ppm: parts per million; sul: sulfadiazine; trim: trimethoprim)

Table S3. Antibiotic resistance gene hits obtained in fishpond-3 and reservoir shotgun metagenomes using using COSMOSID and CARD bioinformatic pipelines. Genes with less than ten hits are not shown.

	COSMOSID characterizations							
	Pool_Jul 15	Pool_Aug 15	Pond_Dec 15	Pond_Mar 16	Res_Jul 13	Res_Aug 13	Res_Jan 14	Res_Mar 14
Sulphonamide sul2	2838	2899	2865	2314	0	0	0	0
Tetracycline tetBP	0	1899	0	0	0	0	0	0
Tetracycline tetRG	328	0	263	284	0	202	175	468
Tetracycline tetE	0	0	1555	0	0	0	0	0
Tetracycline tetS	1468	0	0	0	0	0	0	0
Tetracycline tetG	0	0	0	0	0	0	0	1261
Tetracycline tetR	0	0	1046	137	0	0	0	0
MDR-Efflux-pump mexQ	0	0	963	0	0	0	0	0
Sulphonamide sul1	0	416	321	205	0	0	0	0
MDR-Efflux-pump mexB	0	0	936	0	0	0	0	0
blaOXA 50	0	0	852	0	0	0	0	0
MDR-Efflux-pump mexN	0	0	744	0	0	0	0	0
MDR-Efflux-pump mexP	0	0	631	0	0	0	0	0
Repressor-of-mexJKmexL	404	0	189	0	0	0	0	0
Repressor nalD	175	0	402	0	0	0	0	0
MDR-Efflux-pump mexM	203	0	325	0	0	0	0	0
Trimethoprim dfrD	0	0	0	0	518	0	0	0
Tetracycline tet33	0	0	242	201	0	0	0	0
Outer-membrane-factor oprM	0	0	429	0	0	0	0	0
Suppressor-of-MexTmexS	0	0	360	0	0	0	0	0
Aminoglycoside aph3' IIb	0	0	342	0	0	0	0	0
MDR-Efflux-pump opmH	0	0	294	0	0	0	0	0
Trimethoprim dfrA31	0	0	0	0	282	0	0	0
Membrane-fusion-protein mexA	0	0	274	0	0	0	0	0
Efflux-pump triA	0	0	268	0	0	0	0	0
Sensor-protein soxR	0	86	173	0	0	0	0	0
Beta-lactam-resistance blaACT 60 Branch	0	0	0	138	0	0	0	0
Efflux-pump amrA	0	0	136	0	0	0	0	0
Trimethoprim d 2268 Branch	0	115	0	0	0	0	0	0
Aminoglycoside aadA 2142 Branch	0	0	0	64	0	0	0	0
Beta-lactam-resistance blaIMI 1671 Branch	0	54	0	0	0	0	0	0
Phenicol cmlA1	0	0	54	0	0	0	0	0
Tetracycline tet35	0	0	54	0	0	0	0	0
Beta-lactam amp 2082 Branch	0	0	0	49	0	0	0	0
Beta-lactam-resistance blaOXA 462 Branch	0	0	0	0	0	37	0	0
Trimethoprim dfrA 2390 Branch	0	0	36	0	0	0	0	0
Beta-lactam-resistance blaGOB 5	0	26	0	0	0	0	0	0
Integron-mediated-quinolone-resistance qnrVC5	0	0	0	0	17	0	0	0

Quinolone qnrS2	13	0	0	0	0	0	0	0
Beta-lactam-resistance blaPAO	0	0	0	12	0	0	0	0
Beta-lactam-resistance blaPDC 745 Branch	0	0	0	12	0	0	0	0

CARD characterizations

	Pond_Jul 15	Pond_Aug 15	Pond_Dec 15	Pond_Mar 16	Res_Jul 13	Res_Aug 13	Res_Jan 14	Res_Mar 14
aminocoumarin	575	448	406	428	1629	1135	265	651
mfd	199	169	152	169	753	528	112	311
dfrE	131	133	111	118	450	320	80	249
adeG	1026	12	15	6	14	14	4	24
pmrE	86	85	79	47	306	195	74	144
mexK	240	64	71	72	210	202	57	73
msbA	108	60	40	28	156	155	35	87
mexB	378	51	80	34	32	45	13	12
mexF	228	33	56	58	69	35	18	65
mexW	184	32	74	45	87	64	10	39
macB	57	74	35	35	132	126	22	48
rifampin	46	92	20	11	114	177	12	24
smeE	136	27	44	25	27	55	16	34
mtrA	32	51	19	17	133	67	7	33
rosB	95	50	31	23	61	42	12	21
acrB	141	23	24	19	43	45	9	19
mdtB	64	26	33	24	54	36	10	34
ceoB	165	20	12	7	5	5	1	
mexI	25	16	36	20	38	25	11	20
mexQ	34	15	29	10	49	28	7	16
sav1866	17	14	16	14	42	58	6	21
mdtC	56	13	16	13	31	25	12	16
oqxB	41	12	2	11	42	21	2	24
cpxR	15	8	9	7	50	32	12	18
mdsB	28	23		14	33	17	9	22
tetG	10	2	30	10	30		10	47
sul2	38	38	34	26		1		
acrD	41	12	9	8	18	22	9	13
oleC	10	7	23	13	35	16	8	15
efpA	27	4	3	8	29	31	3	5
mexY	22	8	18	14	17	21		6
bcrA	1	13	8	6	34	17	10	14
mdtF	78	1	9		7	3	2	3
mexD	27	8	20	8	9	12	6	11
tetB(P)	5	30	13	15	12	15	5	5
sul1	17	33	36	9				
arnA	8	3	11	9	30	23	3	4
acrF	33	6	8	5	4	18	5	2
OpmH	48		12	8	1	6	5	1
CRP	4	6	6	8	16	26	1	8
mexN	27	7	12		8	3	1	11

smeR		7	7	7	10	25	4	9
adeJ	10	8	2	20	12	8		5
novA	3	16	3	4	21	7	4	5
bacA	11	5	6		15	15		8
mtrD	12	2	10	4	10	8	5	5
adeB	30	6	7	4	1			6
floR	3		18	4	6	2	5	15
smeB	3	10	13	2	3	11	1	9
mexC	11	1	6	6	7	6	6	5
oprN	29	4	7	8				
efrB	3		3	8	12	15	1	2
rosA	21	4	4	2	7	1	4	1
adeF	42			1				
mexA	15	6	12	7		3		
OXA-36	17	4	4	2	7	6	1	2
TriC	6	11	16	1	4	1		4
kdpE	16	3	1	1	5	12		3
mexL	22		8	6		2		
smeF	19	2	7	3	4	2		
drrA	5		2	1	16	8	1	3
smeD	13	4	3	1	6	4	2	3
nalD	19	1	12	3				
RbpA	14	2	6	3	4	3	1	2
tet33	3		21	8	3			
amrB	1		8	4	7	8	2	4
oprM	14	1	15	1	2		1	
golS	9	7	8	2		3		3
mexE	13	2	10	5	1			
qepA	20	5	1	2	1	1		1
tetT	4	3	3	5	3	1		10
tetA(P)		13	3	6		5		1
cmeB	5		4	2	9	3	2	1
dfrA3	1	1	8		8	4		4
tet35	1	1			5	3	1	15
ceoA								24
emrA	5	3		3	6	4	2	1
mdsC			23			1		
mexV	7	3	11	3				
tetE	2		14	1		2		5
adeH	21				1			1
arlR	1	1	4		6	7		4
mexP	3	5	12	1		1		
OXA-198		2	2	2	2	10		4
mel			1		14	1		5
pp-flo	1	1	7		1		1	10
mexT			1	2	5	7	1	4
OXA-50	3		13	2	2			
patA	2	1	4	3	5	5		
tetM		1	3		1	13		1

tsnr	2	3	1	1	5	6		1
acrA	11	1	1	2	2	1		
OXA-209			12	1	1	1		2
oprJ	3	1	9	1		1		
OXA-12	3	1	1	2	4	4		
pmrB	1	2	6	2		2	1	
qacH	1	1			9			3
tetC	3	1		1	6	1		2
tolC	6				5	1		2
adeI	1	1				11		
emrB	5		3	1	1	1		2
vatF			1		8	4		
opmE	6	1	5					
PmrF	1		1	2	2	6		
tetS	11		1					
tetX				1	6			5
TriA	3	1	5	2			1	
ANT(2")-Ia		1	7	3				
cat	1		1	3	4		1	1
efrA		3		1	3	2	1	1
mexJ	2		2	2		2	1	2
mexM	3	1	6	1				
opcM	9	1			1			
VanRO	8				1			2
AAC(6')-Ib8			2			1	4	3
baeR				2	3	1	3	1
dfrD						10		
mexX	1		2	1			6	
oprA	1	2	1	1	1	3	1	
OXA-205	4	2	4					
OXA-53	3				1	5		1

Table S4. Pearson's correlation coefficients followed by Canonical Correspondence Analysis (CCA) between phyla (with relative abundance >5.0% at any of the sampling profile), ARGs, integrase genes and environmental parameters (temperature and precipitation).

Phylum	Temp	pptn	*int1*	*sul1*	*tetA*	*blaTEM*	*sul2*	*dfrA1*
Proteobacteria FP	-0.698*	0.836**	0.049	-0.387	0.246	-0.405	-0.303	0.104
Actinobacteria FP	-0.076	0.250	0.026	0.158	0.057	0.230	0.465	-0.030
Verrucomicrobia FP	-0.531	0.227	-0.063	-0.062	0.168	-0.030	0.403	0.109
Bacteroidetes FP	-0.402	0.322	0.928**	0.718*	0.869**	0.096	0.135	0.594
Cyanobacteria FP	0.801*	-0.706	0.098	0.542	-0.383	-0.127	0.434	0.260
Planctomycetes FP	0.604	-0.390	-0.275	0.119	-0.435	-0.436	0.002	-0.146

Proteobacteria Res	-0.509	0.553	0.020	-0.023	0.238	-0.195	0.263	0.080
Actinobacteria Res	-0.182	0.436	0.597	0.390	0.429	-0.031	0.305	0.674
Verrucomicrobia Res	-0.535	0.252	-0.009	0.061	0.082	-0.235	0.250	-0.163
Bacteroidetes Res	-0.900**	0.658	0.498	0.092	0.637	-0.151	-0.097	0.247
Cyanobacteria Res	0.896**	-0.660	-0.044	0.373	-0.478	-0.266	0.232	0.196
Planctomycetes Res	0.805*	-0.593	-0.247	0.305	-0.502	-0.212	0.329	-0.073

*Significant at **P** < 0.05, **Significant at **P** < 0.01 (temp: temperature; pptn: precipitation).
